# The impact of senescence-associated T cells on immunosenescence and age-related disorders

**DOI:** 10.1186/s41232-018-0082-9

**Published:** 2018-12-24

**Authors:** Yuji Fukushima, Nagahiro Minato, Masakazu Hattori

**Affiliations:** 1 Department of Immunosenescence, Graduate School of Medicine, Kyoto University, Kyoto, 606-8507 Japan; 2 DSK Project, Medical Innovation Center, Graduate School of Medicine, Kyoto University, Kyoto, 606-8507 Japan

**Keywords:** Age-related disorders, Immunosenescence, Osteopontin, Senescence-associated T cells, Thymus

## Abstract

Immunosenescence is age-associated changes in the immunological functions, including diminished acquired immunity against infection, pro-inflammatory traits, and increased risk of autoimmunity. The proportions of memory-phenotype T cells in the peripheral T cell population steadily increase with age, but the relationship between this change and immunosenescent phenotypes remains elusive. Recently, we identified a minor memory-phenotype CD4^+^ T cell subpopulation that constitutively expressed PD-1 and CD153 as a bona fide age-dependent T cell population; we termed these cells senescence-associated T (SA-T) cells. SA-T cells exhibit characteristic features of cellular senescence, with defective T cell receptor-mediated proliferation and T cell cytokine production. However, upon T cell receptor stimulation, SA-T cells secrete abundant atypical pro-inflammatory cytokines such as osteopontin and chemokines, reminiscent of the SA-secretory phenotype. In addition to aging, SA-T cells accumulate and cause persistent inflammation in tissues following a wide range of insults including immune complex deposition, metabolic stresses, vascular damages, and tumors. In this review, we summarize the recent understanding of immunosenescence with particular focus on SA-T cells and their role in various age-related disorders.

## Background

**Fig. 1:**
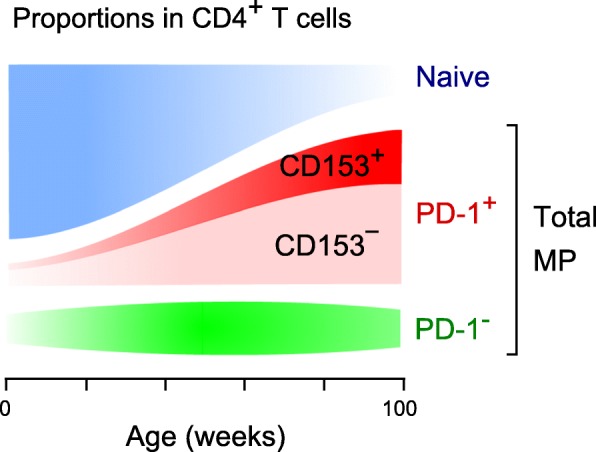
Increase in MP CD4^+^ T cells with age. Proportion of MP T cell subsets or total MP CD4^+^ T cell fraction in the spleens of female B6 mice are shown

Aging processes affect diverse aspects of tissue functions as well as their functional networks such as the immune system. Age-associated changes in immune functions, collectively called immunosenescence, are characterized by diminished adaptive immune competence leading to reduced infection resistance, pro-inflammatory traits that may underlie chronic inflammatory disorders, and increased risk of autoimmunity in the elderly [1, 2]. Age-related changes may occur in most types of cells in both innate and adaptive immune systems, but the exact mechanism of immunosenescence remains largely elusive. The most dramatic change in the adaptive immune system is the involution of the thymus, the sole organ devoted to generation of T cells, which causes a progressive reduction in the output of naïve T cells with age [3]. Homeostatic proliferation (HP) of naïve (CD44^low^ CD62L^high^) T cells results in the phenotypic conversion to memory-phenotype (MP) (CD44^high^) T cells [4]. Hence, the age-dependent increase in the proportions of MP T cells can be attributed to the increasing T cell HP, which compensates for decrease in T cell output due to physiologic thymic involution, rather than to antigen-driven immune responses [3]. MP CD4^+^ T cells, which become predominant with age, tend to exhibit impaired T cell receptor (TCR)-mediated proliferation and IL-2 production [5, 6]. We recently identified a distinct subpopulation that constitutively expressed PD-1 and, to a lesser extent, CD153, in the MP CD4^+^ T cells of aged mice (Fig. 1). We believe that these PD-1^+^/CD153^+^ MP CD4^+^ T cells represent a bona fide age-dependent T cell population with classical features of cellular senescence; accordingly, we refer to them as SA-T cells [7, 8].

### Cellular and functional features of SA-T cells

**Fig. 2:**
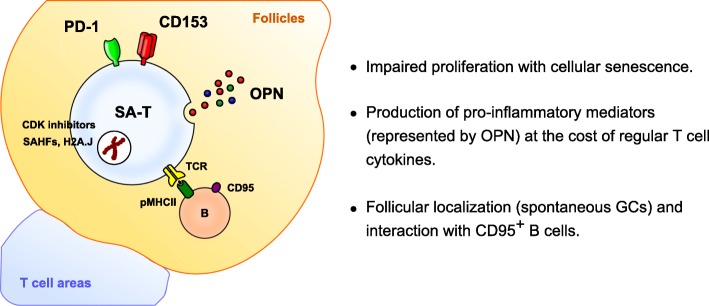
Properties of SA-T cells

The TCR responsiveness of the overall CD4^+^ T-cell population, in terms of proliferation and regular cytokine production, diminished gradually with age. Our careful studies, however, revealed that these effects were attributed primarily to the increase in the proportions of SA-T cells with age, given that the residual naïve and PD-1^−^ (CD153^−^) MP CD4^+^ T cells in aged mice exhibited TCR responsiveness comparable to those from young mice [7]. The defective proliferation of SA-T cells could be attributed to cellular senescence, in that these cells exhibited a marked increase in the expression of SA-cell cycle inhibitors (*Cdkn1a* and *Cdkn2b*), SA-heterochromatin foci (SAHFs), and SA-β-galactosidase (Fig. 2). Senescent cells tend to resist apoptosis; consistent with this, SA-T cells were quite stable over long-term culture [8], probably accounting for the progressive accumulation of SA-T cells with age despite their defective proliferation capacity. Recent reports showed that a micro-RNA, miR-181a, is a T cell-specific senescence indicator that enhances TCR signal strength [5, 9], and indeed, SA-T cells exhibited a remarkably reduced expression of miR-181a [8].

Although SA-T cells were defective in production of regular T cell cytokines such as IL-2 and IL-4, upon TCR stimulation they produced abundant pro-inflammatory cytokines and chemokines such as osteopontin (OPN), IFN-γ, Ccl3, and Ccl4 (Fig. 2) [8]. Senescent cells are metabolically active, and senescent mesenchymal and endothelial cells spontaneously secrete a wide range of bioactive proteins, in particular inflammatory cytokines and chemokines, collectively termed the SA-secretory phenotype (SASP) [10]. In SA-T cells, it appears that the expression of the SASP factors is somehow linked to TCR signaling. Interestingly, despite the high expression of PD-1, OPN production by SA-T cells upon TCR stimulation was not inhibited at all by concomitant PD-L1 stimulation, whereas this treatment significantly inhibited the production of IL-4 [8]. Also, the TCR-induced OPN secretion, but not IL-4 production, of SA-T cells was significantly enhanced by CD153 co-stimulation. The engagement of CD153 on innate immune cells induces the production of pro-inflammatory mediators such as IL-6, IL-8, Ccl2, Ccl3, and Ccl4 [11, 12]. Thus, the SASP of SA-T cells seems to be triggered by an alternative, PD-1 signal-resistant pathway downstream of TCR signaling. A recent study showed that the SASP in senescent fibroblasts is associated with expression of a rare histone variant, H2A.J [13], and we confirmed that SA-T cells exhibit the expression of H2A.J [Fukushima, unpublished observation].

SA-T cells are abundant throughout the lymphohematopoietic organs of aged mice, including the spleen, lymph nodes, and bone marrow [7]. Of note, SA-T cells are preferentially localized in the follicular region of white pulp in the spleen, where B cells reside, often in association with germinal centers (GCs) that spontaneously develop as mice age (Fig. 2) [8]. In agreement with this finding, B cell-deficient (μMT) mice develop minimal SA-T cells with age, indicating that the development of SA-T cells is dependent on the presence of B cells [8]. Among B cells, SA-T cells interact most efficiently with CD95^+^ B cells such as GC-B cells and so-called age-associated B cells (ABCs) [14]. Although several features of SA-T cells such as high expression of PD-1, Cxcr5, and Bcl6 resemble those of T follicular helper (T_FH_) cells, which develop in association with GCs during the antigen-driven immune response [15], T_FH_ cells show no evidence of cellular senescence or CD153 expression and are thus distinct from SA-T cells. In lupus-prone BWF_1_ mice that robustly develop GC reactions [8], SA-T cells (PD-1^+^ CXCR5^low^ CD153^+^) and T_FH_ cells (PD-1^+^ CXCR5^high^ CD153^−^) are detected in distinct fractions [16]. Of note, the SA-T cells were hardly detected in the circulation in aged and lupus-prone mice [8], whereas these cells are markedly increased in the aged and chronically inflamed tissues in humans and mice (see below). Thus, although expression of several markers such as NK-related markers is reported to increase with age in CD4^+^ T cells of human peripheral blood, their possible relation to SA-T cells remains to be seen.

### Mechanisms of SA-T cell generation

The age-dependent increase in SA-T cells could be due to CD4^+^ T cell-intrinsic effects or to the tissue environment of aged individuals. We found that the naïve CD4^+^ T cells transferred from young mice robustly proliferated in an aged host environment and underwent significant conversion to SA-T cells, whereas in young hosts, the same T cells barely proliferated and generated few SA-T cells [17]. Thus, the aged, but not young, host environment plays a crucial role in the development of SA-T cells from naïve CD4^+^ T cells. Similar results were observed under the experimental T-lymphopenic conditions, such as γ-ray-irradiated mice and *CD3ε*^*−/−*^ mice [17], suggesting that sustained antigen-independent T cell HP underlies the development of SA-T cells [17]. Thymectomy at an early age significantly accelerated the increase in SA-T cells, whereas implantation of embryonic thymus attenuated the increase and accumulation of SA-T cells with age [17]. Hence, a major force driving the increase in HP and resulting generation of SA-T cells in aged mice is the decreased output of naïve T cells from the thymus [3, 18]. The HP of peripheral naïve CD4^+^ T cells is driven by self-peptide/MHC-II on B cells and dendritic cells, along with the homeostatic cytokines such as IL-7 and IL-15 [4]. While antigen-driven CD4^+^ T cell proliferation during an immune response is followed by differentiation into effector cells and cessation of cell proliferation, HP in the acute lymphopenia is not associated with effector differentiation [19]. Among CD4^+^ T cells undergoing HP, SA-T cells are confined to those that experienced extensive (> 8) cell divisions [17]. This observation suggests that replicative senescence due to sustained cell divisions during HP is involved in generation of SA-T cells.

CD4^+^ T cell proliferation is exclusively fueled by oxidative phosphorylation in mitochondria, regardless of antigen-driven or homeostatic proliferation, whereas effector differentiation in the former is associated with a shift of energy metabolism to aerobic glycolysis [19]. Cellular energy metabolism plays a role in controlling cellular senescence [20], and metabolic stresses via sustained oxidative phosphorylation during continued HP may also promote the development of SA-T cells. In agreement with the notion, treatment of lupus-prone mice, in which SA-T cells play a crucial role in pathogenesis (see below), with a combination of mitochondrial and glucose metabolism inhibitors remarkably attenuates the increase in PD-1^+^ MP CD4^+^ T cells and ameliorates the severity of lupus [21]. In addition, a recent report revealed the role of Menin-Bach2 in the CD4^+^ T cell senescence, suggesting the involvement of epigenetic regulation [22].

The numbers of GC-B cells as well as ABCs are increased with age. Because CD95^+^ GL7^+^ GC-B cells and CD95^+^ GL7^−^ B cells, probably including ABCs, show the most efficient antigen-presenting function to the SA-T cells among B cell populations [8], the age-dependent increase in GC-B cells and ABCs may contribute to the increase and accumulation of SA-T cells with age. Toll-like receptor 7 (TLR7) plays an essential role in spontaneous development of GCs and autoimmunity in lupus-prone mice [23]. We found that administration of the ligand for TLR7, but not TLR3 or TLR4, caused a robust increase in SA-T cells in normal mice, in concordance with the increase in GC-B cells [16]. TLR7 is a receptor for single-stranded RNA expressed on several types of immune cells, including B cells, and stimulates the proliferation of GC-B cells and ABCs [14, 24]. Hence, it seems likely that TLR7 ligands induce the increase in SA-T cells indirectly through the activation of GC-B cells and ABCs.

### SA-T cells in diseases

In addition to the chronological aging, accumulating evidence indicates that the SA-T cells are markedly increased in the tissues under persisted inflammation, often in association with the tertiary lymphoid tissues, of chronic inflammatory disorders.

#### SLE

**Fig. 3:**
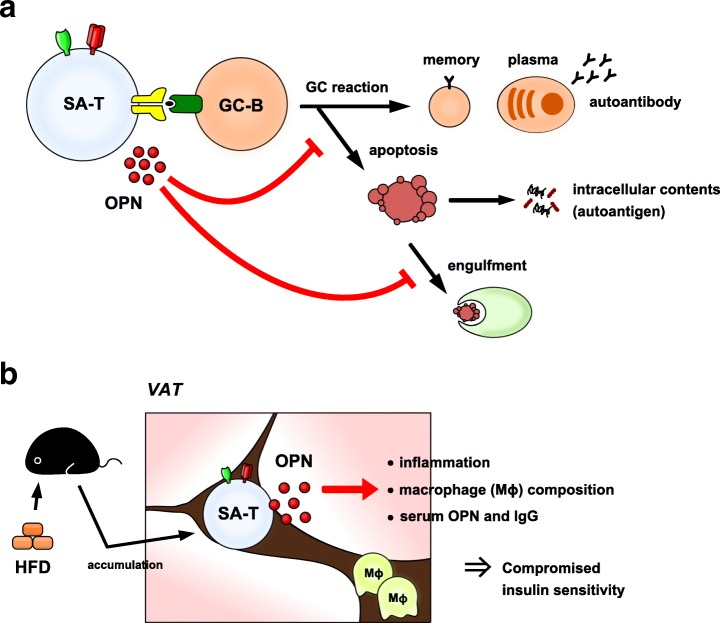
Involvement of SA-T cells in the pathogenesis of age-related disorders. **a** SLE. **b** Chronic inflammation in VAT

Systemic lupus erythematosus (SLE) is a female-dominant systemic autoimmune disease characterized by the development of a wide variety of autoantibodies including anti-nuclear antibodies, which are deposited as immune complexes in tissues such as kidney glomeruli, where they cause chronic nephritis [25]. The disease is associated with remarkable development of spontaneous GCs [26]. In lupus-prone female NZB/W F_1_ (BWF_1_) mice, we discovered that PD-1^+^ CD153^+^ SA-T cells are robustly elevated in association with the development of spontaneous GC reactions as the disease progresses [8]. These effects are reminiscent of changes observed in normal-aged mice but occur much earlier and more robustly in genetically lupus-prone mice. Such SA-T cells are apparently autoreactive, in that they secrete large amounts of OPN in response to autologous GC-B cells in a TCR- and MHC-II-dependent manner [8]. Accelerated T cell HP underlies systemic autoimmunity via an enriched T cell population with higher intrinsic reactivity to self-peptides/MHC-II [27, 28], and we have confirmed that the robust increase in SA-T cells in female BWF_1_ mice occurs in association with a remarkable increase in endogenous CD4^+^ T cell proliferation [8]. Involvement of OPN has long been implicated in the pathogenesis and clinical manifestations of human SLE [29]. We have shown that OPN promotes the autoreactive GC-B cell development in two ways (Fig. 3a). First, it inhibits B cell receptor-induced GC-B cell apoptosis [8]. Second, it interferes with the engulfment of resultant apoptotic GC-B cells by tingible-body macrophages by inducing sustained Rac1 activation, leading to the impaired disposal of nuclear autoantigens [16]. Consistently, administration of neutralizing anti-OPN antibody to female BWF_1_ mice significantly ameliorates the progression of lupus nephritis [8]. Thus, SA-T cells play an important role in lupus pathogenesis by secreting abundant OPN in spontaneous GCs, thereby promoting autoantibody production.

#### Tissue stresses and chronic inflammatory disorders

Various metabolic stresses that cause chronic low-grade inflammation may also drive cellular senescence [30]. A high-fat diet (HFD) causes obesity with chronic inflammation in visceral adipose tissues (VATs) and predisposes humans to metabolic and cardiovascular diseases [31]. We have found that under HFD, but not a normal diet, the VATs exhibit a remarkable accumulation of PD-1^+^ CD153^+^ SA-T cells that secrete abundant OPN [32], which plays an important role in obesity-induced adiposity and insulin resistance (Fig. 3b) [33]. Cell transfer analysis has revealed that SA-T cells are primarily responsible for the initiation of VAT inflammation and the development of insulin-resistance [32]. The mechanism of SA-T cell accumulation in the VATs under HFD remains to be determined. However, because SA-T cells preferentially increase in the VATs (comparison with other tissues), under HFD, it is possible that persistent metabolic stresses in visceral adipocytes play a role. Notably, such SA-T cells persist over the long term in VATs, even after the HFD was discontinued and the body weight became normalized [34], consistent with the stability of SA-T cells [8]. We previously reported that PD-1^+^ CD153^+^ SA-T cells infiltrate and accumulate in the kidneys with marked immune complex deposition in lupus mice, often forming tertiary lymphoid tissues in the kidney parenchyma [8]. Thus, it seems that, in addition to autoantibody production in splenic GCs, SA-T cells may also be involved in the progression of kidney inflammation in lupus. A more recent report indicated that various tissue insults in kidney, such as transient vascular occlusion, urethral obstruction, and nephrotoxic drugs, result in progression of aggravated nephritis with dense tertiary lymphoid tissues in aged, but not young, mice, in a CD4^+^ T cell-dependent manner [35]. All in all, these findings suggest that CD4^+^ T cell senescence is associated with various tissue stresses, and the resultant accumulation of SA-T cells in insulted tissues may lead to persistent tissue inflammation and dysfunction.

#### Cancer

The growth of malignant cells with distinct metabolic patterns [36] represents a potent insult to a tissue and may profoundly affect the functions of cells within that tissue, including immune cells. Tumor-infiltrating T cells exhibit dysfunctions including anergy, exhaustion, and senescence, allowing cancer cells to evade host immunity [37]. Accumulating evidence indicates that expression of PD-1 plays an important role in such T cell dysfunction. Persistent PD-1 signaling in effector CD8^+^ T cells causes profound alterations of energy metabolism, eventually leading to TCR unresponsiveness [38, 39]. We previously reported that the abundance of CD4^+^ T cells constitutively expressing PD-1 was robustly increased in lymphohematopoietic tissues during the progression of leukemia, leading to the profound immunodepression [7]. Leukemia-associated CD4^+^ T cells exhibit characteristics to those of SA-T cells occurring in normal aged mice, including senescence features [7], indicating that systemic leukemia causes a rapid progression of CD4^+^ T cell senescence. It has long been known that aggressive inflammation of cancer tissues often predisposes to cancer progression [40]. Hence, the increase in SA-T cells bearing potent inflammatogenic activity in tumor tissues may have a significant impact on cancer growth. The risk of cancer development increases with age, and the involvement of immunosenescence in this process has been an issue of concern. Notably, a recent study indicated that genetic ablation of tissue-resident senescent cells significantly prolongs the lifespan of mice, which also exhibit lower rates of cancer death [41]. Thus, T cell senescence may contribute to cancer development and progression [42].

## Conclusion

Increasing evidence supports an important role for immunosenescence in diverse age-related chronic disorders and cancer. SA-T cells represent one of the first T cell populations shown to exhibit the classical features of cellular senescence, including defective cell proliferation and the SASP. SA-T cells accumulated with age, accounting for the major phenotypes of immunosenescence. The SASP of SA-T cells is linked to TCR signaling, conferring a unique and potent inflammatogenic activity in response to antigens. Extensive cell divisions during increasing HP with age underlie the age-dependent increase in SA-T cells. A variety of tissue stresses may also promote the development of SA-T cells and their accumulation in affected tissues. As such, SA-T cells are involved in chronic inflammation in the tissues under various stresses, such as immune complex deposition, metabolic and vascular insults, and possibly cancer. Recent evidence indicates that the selective elimination of tissue senescent cells leads to a significant improvement of age-associated tissue dysfunctions with prolonged lifespan. Consequently, tissue senescent cells are emerging as a crucial target for preventive and therapeutic intervention of age-related chronic disorders. Targeted elimination of SA-T cells represents a promising strategy for controlling chronic inflammatory disorders and possibly cancer.

## Abbreviations

ABCs: Age-associated B cells
BWF_1_: NZB/W F_1_
GC: Germinal center
HFD: High-fat diet
HP: Homeostatic proliferation
MP: Memory-phenotype
OPN: Osteopontin
SAHFs: SA-heterochromatin foci
SASP: SA-secretory phenotype
SA-T: Senescence-associated T
SLE: Systemic lupus erythematosus
TCR: T cell receptor
T_FH_: T follicular helper
TLR: Toll-like receptor
VATs: Visceral adipose tissues
